# High-Throughput Cell Trapping in the Dentate Spiral Microfluidic Channel

**DOI:** 10.3390/mi12030288

**Published:** 2021-03-09

**Authors:** Jiawei Lu, Bo Dai, Kan Wang, Yan Long, Zhuoqing Yang, Junyi Chen, Shaoqi Huang, Lulu Zheng, Yongfeng Fu, Wenbin Wan, Songlin Zhuang, Yangtai Guan, Dawei Zhang

**Affiliations:** 1 Engineering Research Center of Optical Instrument and System, the Ministry of Education, Shanghai Key Laboratory of Modern Optical System, University of Shanghai for Science and Technology, Shanghai 200093, China; 193730599@st.usst.edu.cn (J.L.); daibo@usst.edu.cn (B.D.); 193730605@st.usst.edu.cn (Y.L.); 202430406@st.usst.edu.cn (J.C.); shaoqihuang19960720@gmail.com (S.H.); llzheng@usst.edu.cn (L.Z.); slzhuang@yahoo.com (S.Z.); 2 Department of Neurology, Renji Hospital, School of Medicine Shanghai Jiaotong University, 160 Pujian Rd, Shanghai 200127, China; 21221@renji.com (K.W.); wanwenbin026725@renji.com (W.W.); 3 National Key Laboratory of Science and Technology on Micro/Nano Fabrication, School of Electronics Information and Electrical Engineering, Shanghai Jiao Tong University (SJTU), Shanghai 200240, China; yzhuoqing@sjtu.edu.cn; 4 Department of Medical Microbiology and Parasitology, School of Basic Medical Sciences, Fudan University, Shanghai 200032, China; yffu@fudan.edu.cn

**Keywords:** cell trapping, spiral channel, dentate structure, electric circuit analogy

## Abstract

Cell trapping is a very useful technique in a variety of cell-based assays and cellular research fields. It requires a high-throughput, high-efficiency operation to isolate cells of interest and immobilize the captured cells at specific positions. In this study, a dentate spiral microfluidic structure is proposed for cell trapping. The structure consists of a main spiral channel connecting an inlet and an out and a large number of dentate traps on the side of the channel. The density of the traps is high. When a cell comes across an empty trap, the cell suddenly makes a turn and enters the trap. Once the trap captures enough cells, the trap becomes closed and the following cells pass by the trap. The microfluidic structure is optimized based on the investigation of the influence over the flow. In the demonstration, 4T1 mouse breast cancer cells injected into the chip can be efficiently captured and isolated in the different traps. The cell trapping operates at a very high flow rate (40 μL/s) and a high trapping efficiency (>90%) can be achieved. The proposed high-throughput cell-trapping technique can be adopted in the many applications, including rapid microfluidic cell-based assays and isolation of rare circulating tumor cells from a large volume of blood sample.

## 1. Introduction

Cell trapping is a technique to isolate single cells or cell clusters of interest from a large group of cells and immobilize the target cells at a specific location for further cellular analysis. Cell trapping technique plays an important role in the various biomedical diagnosis and research fields, including cell-based assay, cellular heterogeneity analysis, cell-to-cell signaling study, drug screening and rare circulating tumor cell monitoring [1,2,3,4,5,6,7]. A diversity of cell trapping techniques have been demonstrated. In general, the cell-trapping techniques can be grouped into two categories based on cell-driven mechanisms, i.e., active trapping that applies an external force field to manipulate cells and passive trapping that harnesses a hydrodynamic effect to drive cells in the microfluidic structure.

Active trapping employs optical, electrical, acoustic or optothermal technologies to generate a force for retrieving and stopping cells [8,9,10]. Dielectrophoretic trapping utilizes negative charges of cell-membrane proteins to push cells away from electrodes (negative dielectrophoresis) or pull cells toward electrodes (positive dielectrophoresis) [11,12]. Electrode arrays can be designed to pattern thousands of traps and each trap with single-cell resolution can be realized. The dielectrophoretic force should be large to balance hydrodynamic drag force, hydrodynamic lift force and gravity. It is worth noting that the strength of dielectrophoresis is a key factor to determine the maximum flow rate. In a typical dielectrophoretic trapping platform, the maximum flow rate for trapping particles with diameter about 20 μm is no more than 30 μL/min [13]. Besides, the optical tweezer, which has been developed into scientific instruments, is a powerful tool for particle or cell transportation, retrieving and trapping [14,15]. Similar to dielectrophoretic trapping, an optical tweezer also harnesses the dielectric property of cells. A strong electric field gradient exists in a focused laser beam. Cells can be attracted along the gradient to the strongest electric field. To achieve multiple-cell trapping, an optical trap array can be realized by using a micro-mirror array or a microlens array to generate multiple focusing spots [16,17]. High optical power results in a strong optical force and can efficiently balance the hydrodynamic drag force, which benefits particle trapping in a high-flow-rate flow. But high-power light-induced damage might cause cellular lysis and affect growth and cell division ability [18]. Thus, optical trapping should operate at a relatively low and safe power range, but it is hard to sustain cells in the trap where the flow rate is high and the throughput is limited. For example, an on-chip optical tweezer, which is based on a gradient refractive index lens, is capable of efficiently trapping and flexibly manipulating single cells with a diameter ranging from 10 μm to 15 μm [10]. The maximum flow rate cannot exceed 400 nL/min. In addition, surface acoustic wave (SAW) technology provides an alternative way for cell trapping [19,20,21]. SAW exerts an acoustic radiation force to drive and trap cells. The required power density of SAW for cell trapping is lower than that of the optical tweezer and the dielectrophoretic tweezer and the acoustic tweezer is regarded as a less invasive tool for cell manipulation [22]. Even if the operation is under a low acoustic power, an SAW tweezer can drive cells in the flow with an extremely high flow rate (up to 500 μL/min [23]). However, it is hard for an SAW tweezer to simultaneously isolate a large number cells into different traps. There is no doubt that all these active trapping methods are flexible tools for cell transportation and trapping.

Passive trapping captures cells based on hydrodynamics in special microfluidic structures. In most cell-trapping structures, traps are distributed in the way of the flow to intercept the incoming cells [24,25,26,27,28,29]. Each trap has an entrance to let the cells enter the trap and a tiny cavity to hold the cells. The size of the concavity determines the type and the amount of the cells to isolate. Cells flow along the channel towards the trap arrays. Once the cells enter the trap, the cells are stranded in the traps because the hydraulic pressure enforces the cells to stay in the traps. The traps located in the way of the flow can intercept cells, but the shape and the distribution of the traps should be precisely designed to avoid disrupting the flow and to optimize the trap efficiency. Usually, the flow rate for cell trapping in these kinds of trapping platforms is less than 20 μL/min. If the flow rate is too high, the trapped cells, which face the incoming flow, suffer high shearing. Additionally, traps can also be designed out of the way of the main flow to guarantee a high throughput [30,31,32,33]. The main concern of this kind of trapping structure is that it requires a mechanism to efficiently attract cells into the traps. Traps can be placed on the side of a main channel and connect to an auxiliary structure. The pressure drop through the trap between the main channel and the auxiliary structure enforces cells in the main channel to enter the traps. Since the traps are out of the way of the main stream, high-throughput operation can be achieved. Nevertheless, the complex structure results in low density and a limited number of traps. In the inertial microfluidics employing vortex technology, there are two reservoirs, functioning as traps, on the two sides of the main channel [34,35]. On the reservoirs, large cells, experiencing a shear-gradient lift force, are pushed away from the main channel into the reservoirs. Then, the cells continuously orbit in the vortices generated in the reservoirs. The inertial microfluidic can realize size-selective cell sorting and a single-cell assay. Since the cells trapped in the reservoirs rapidly obit within the vortices, it is not convenient to directly observe the static status of the cells in the traps.

In this paper, we propose a dentate spiral microfluidic structure for cell trapping. A main channel spirally connects an inlet and an outlet. Dentate blocks locate on the side of the main channel one by one, forming an array of traps. The gap between the dentate blocks is to immobilize the cells and the concavity in the trap is to store the cells. The influence of the structural design over the flow is analyzed in the simulation. Finally, we demonstrate cell trapping of 4T1 mouse breast cancer cells in the microfluidic chip.

## 2. Materials and Methods

### 2.1. Design of the Dentate Spiral Channel

**Figure 1:**
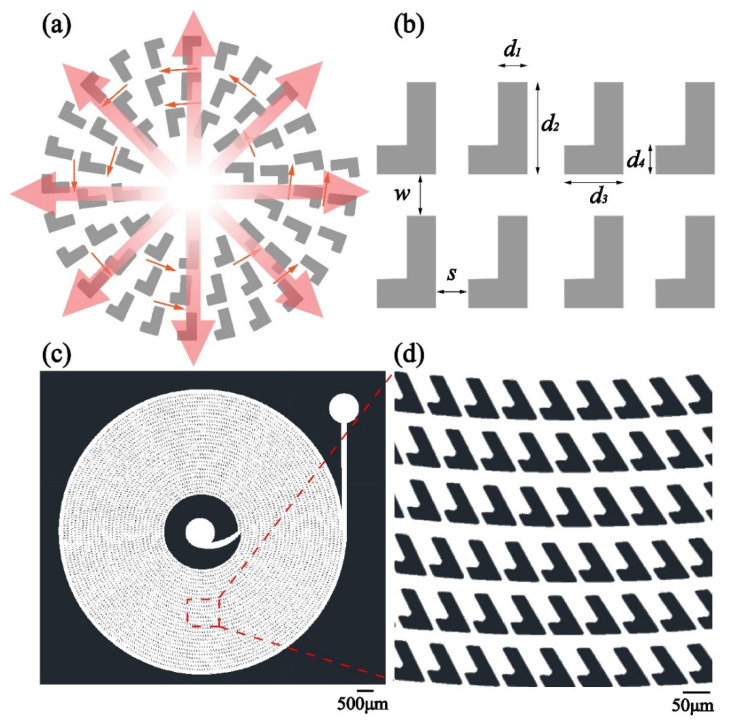
(**a**) Schematic diagram of the dentate spiral microfluidic channel. (**b**) The structure of the “L”-shaped dentate blocks. (**c**) Design of the microfluidic pattern. (**d**) The structure of a group of the dentate blocks, forming the traps.

The cell-trapping structure has a spiral main channel, as shown in Figure 1a. The wall of the channel consists of an array of “L” shaped dentate blocks which are spirally arranged. The separation of an adjacent two blocks is at the scale of a sub-micrometer, which is smaller than the diameter of a cell. There is a concavity in between the two adjacent blocks, forming a trap. Fluid is fed into the structure from the center of the spiral. On the one hand, since the spiral main channel is wide enough, the fluid keeps flowing along the channel until it exits the channel. On the other hand, the size of the gaps in the wall is proper for permeation but is not large enough to allow the cells to pass through. Thus, a portion of the fluid radially permeates outwards from the center through the gaps and the cells in the fluid are trapped in the concavities in the wall.

The model of a part of the structure is depicted in Figure 1b. The design of the microfluidic pattern for cell trapping is illustrated in Figure 1c,d. The width of the main channel, *w*, is 30 μm. The separation of the adjacent blocks, *s*, is 10 μm. The ratio between the width of the channel and the separation of the blocks is optimized, which is discussed in the following section. The dimensions of the dentate blocks are as follows: *d*_1_ = 30 μm, *d*_2_ = 90 μm, *d*_3_ = 60 μm and *d*_4_ = 30 μm. An inlet is in the center of the spiral and an outlet is outside the spiral structure. The entire cell-trapping structure has the diameter of 8 mm and the central internal area for the inlet has the dimeter of 2 mm. There are in total 5304 traps in the designed pattern.

### 2.2. Fabrication of the Microfluidic Chip

**Figure 2:**
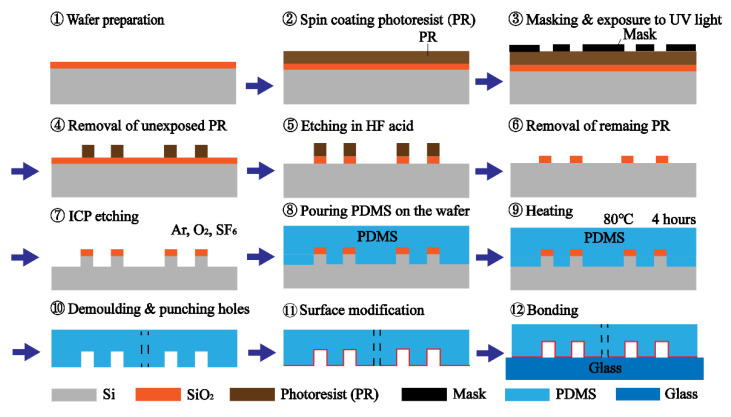
Fabrication procedure of the cell-trapping microfluidic chip.

Figure 2 shows a schematic of the fabrication procedure. The mold of the microfluidic pattern was realized on a SiO_2_/Si substrate by photolithography. A layer of 30 μm photoresist was spin coated on the SiO_2_/Si wafer. A chrome photomask plate deposited with the microfluidic pattern was used in the photolithography. After UV exposure for 8 s, the photoresist exposed by the UV light was removed by the developer. Then, SiO_2_, which was not covered by the photoresist, was etched away by hydrofluoric (HF) acid. The remaining photoresist was removed by dimethyl sulfoxide (DMSO) at 80 °C. Si uncovered by the SiO_2_ was etched by inductively coupled plasma (ICP) etching using a mixture of sulphur tetra fluoride (SF_6_), oxygen (O_2_) and argon (Ar). The etch depth was 35 μm, which corresponded to the height of the microfluidic channel. After cleaning the wafer, the mold was ready for replicating the microfluidic pattern.

In the fabrication of the microfluidic chip, liquid-state polydimethylsiloxane (PDMS), consisting of silicone elastomer and a curing agent at a weight ratio of 10:1 (Sylgard 184, Dow Corning, CA, USA), was poured onto the wafer for casting the structure. After bubble removal in the vacuum machine and heating at 80 °C for 4 h in the oven, the PDMS was solidified. The PDMS layer was peeled off from the wafer and cut into small pieces. After that, the holes for an inlet and an outlet were punched. The surface of the PDMS layer was treated in the air plasma for 1 min. Finally, the PDMS layer was bonded to a slide glass.

**Figure 3:**
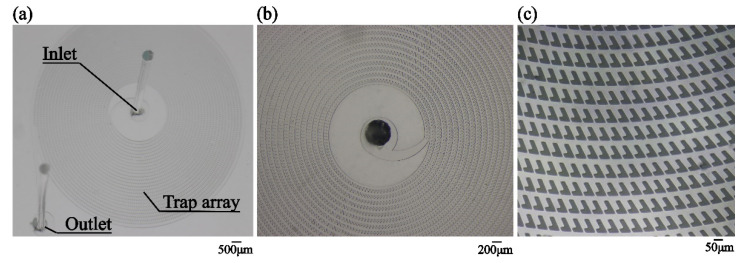
(**a**) Photo of the microfluidic pattern. (**b**) Microscopic image of the center of the microfluidic pattern. (**c**) Microscopic image of a group of cell traps.

Figure 3 illustrates the fabricated microfluidic structure. The spiral main channel connects the inlet and the outlet. The dentate blocks are regularly distributed on the two sides of the main channel. The concavities and the 10 μm separations between the blocks, which are used for cell trapping, can be clearly observed.

### 2.3. Cell Culture and Preparation

4T1 mouse breast cancer cell line (Institute of Biochemistry and Cell biology, Shanghai Institutes for Biological Sciences, Chinese Academy of Sciences) was cultured in Dulbecco’s modified Eagle medium (DMEM) culture medium supplemented with 10% fetal bovine serum (FBS), 100 U/mL penicillin and 100 μg/mL streptomycin. The cells were grown in a 5% carbon dioxide (CO_2_) in a humidified incubator at 37 °C until 70–80% confluence.

## 3. Results

### 3.1. Operation Principle of the Cell Trapping

**Figure 4:**
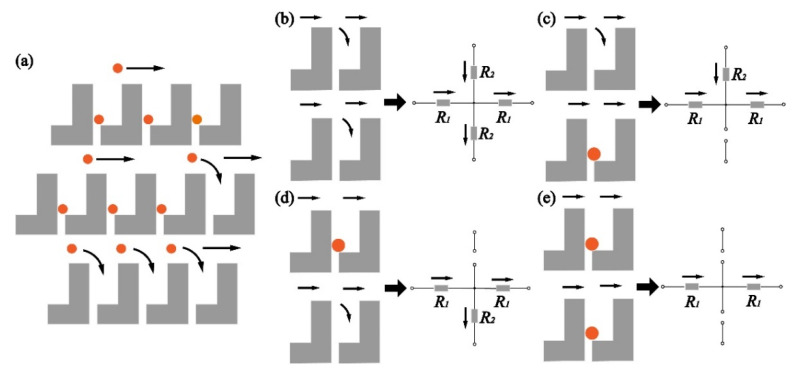
(**a**) Operation principle of the cell trapping in the dentate channel. (**b**–**e**) Equivalent circuits of the microfluidic pattern.

Figure 4a shows the operation principle of the dentate structure. The flow carrying particles flows in the main channel. If the trap is empty, a portion of flow enters the trap and the particle in the flow blocks the gap. Once the gap is blocked, the flow passes by the trap and keeps flowing.

Figure 4b–e depict the equivalent circuit for the microfluidic structure. The inlet and the outlet are analogous to a voltage source and a ground terminal. The pressure drop between the inlet and the outlet can be regarded as a voltage drop from the voltage source to the ground terminal. The flow, corresponding to the current, is from the inlet (the voltage source) to the outlet (the ground terminal). According to the law of mass conservation (Kirchhoff’s current law), the flow (current) coming across an empty trap is divided into two; one keeps flowing in the main channel and the other enters the trap, as shown in Figure 4b. In both the branches, hydraulic resistance exists and affects the flow. The model of the hydraulic resistance is illustrated in Figure S1. The hydraulic resistance [36,37] in the main channel around the trap can be expressed as

(1) $$R_{1}=\frac{12\mu \left(s+d_{3}\right)}{h^{3}w}$$

where *μ* is the viscosity of the fluid and *h* is the height of the channels. The hydraulic resistance in the empty trap can be written as

(2) $$R_{2}=\frac{12\mu \left(d_{2}-d_{4}\right)}{h^{3}\left(s+d_{3}-d_{1}\right)}+\frac{12\mu d_{4}}{h^{3}s}$$

Once the particle enters the trap and blocks the gap, the hydraulic resistance in the trap is extremely high. The equivalent circuit of the trap becomes an open circuit. The flow does not enter the trap and the permeation through the gap is stopped.

**Figure 5:**
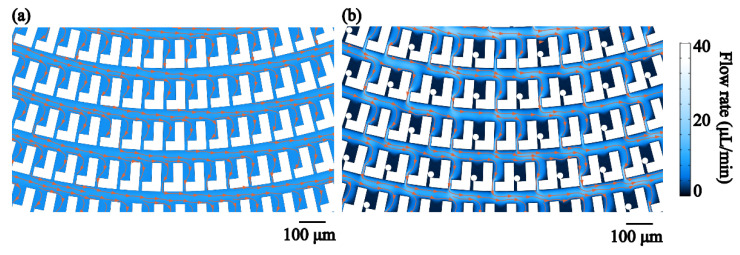
Simulation of the particle trapping. (**a**) The traps are empty. (**b**) Some traps capture the particles. Orange lines indicate the stream lines and the direction of the flow.

A 2D model of the microfluidic structure is established, as shown in Figure S2. The structure has 2339 traps. The gap is 10 μm and the width of the main channel could be flexibly changed. The source is set at the center of the spiral and the drain is at the end of the spiral. The single-phase laminar flow is analyzed. The simulation is conducted using Microfluidics Module in the COMSOL Multiphysics software. Figure 5a shows the simulation result when all the traps are empty. The width of the main channel is 30 μm. The flow rate is 40 μL/min. The distribution of the flow rate is almost uniform in the entire structure. The stream lines show the flow direction. It indicates that the flow enters all the traps. Figure 5b shows the scenario that some gaps are blocked by the particles. The circles representing the particles are sketched in the traps to block the gaps. The diameter of the particles is 16 μm. The flow rate turns to null in the traps where the particles block the gaps. Since every trap is individually isolated, trapping has a very low influence over the flow in the main channel. The flow rate in the main channel is still high in contrast to that in the scenario that all the traps are empty.

Furthermore, the pressure at different positions in the dentate spiral channel is calculated. Figure S3 demonstrates the pressure measurement in the spiral channel radially from the center to the edge. The pressure at the inlet is highest. No matter how the traps are occupied, the pressure drops linearly along the main channel. Since the trap is in between the two adjacent channels, there is a differential pressure between the two sides of the trap. The differential pressure for all the traps are similar. It hints that the force applied on the cells stranded in the traps would be almost identical wherever the traps are.

### 3.2. Optimization of the Microfluidic Structure

**Figure 6:**
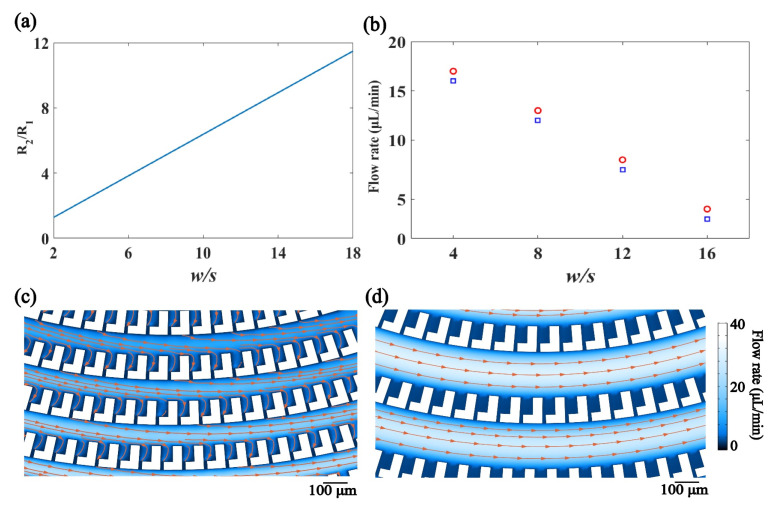
(**a**) The influence of the ratio between the size of the main channel and the gap, i.e., *w/s*, over the ratio between the hydraulic resistance in the trap and the main channel. (**b**) The flow rate through the traps for different values of *w/s*. Red circle: the trap close to the center of the spiral. Blue square: the trap close to the edge of the spiral. (**c**) Simulation of the flow in the microfluidic structure when *w/s* is 8. (**d**) Simulation of the flow in the microfluidic structure when *w/s* is 16.

When encountering a trap, the flow is divided into two. The division of the flow is proportional to the ratio between the hydraulic resistance in the trap and the main channel. Relative high hydraulic resistance leads to a low volumetric flow rate. The ratio of the hydraulic resistance is related to the microfluidic structure. More specifically, the ratio of the hydraulic resistance is determined by the width of the main channel, *w*, and the gap in between the dentate blocks, *s*, as shown in Figure 6a. When the dimension of the main channel is larger than that of the gap in the trap, the hydraulic resistance in the main channel is lower in contrast to that in the trap, resulting in a high flow rate in the main channel. Figure 6b depicts the flow rate through the traps for microstructure with a fixed dimension of the gap and a different width of the main channel. It is obvious that the dimension of the main channel affects the flow division. The flow rate through the traps drops with the increase of the width of the main channel. It hints that if the main channel is wide, where the hydraulic resistance is relatively low, a majority of the flow is in the main channel and only a little flow enters the traps.

Figure 5a and Figure 6c,d demonstrates the flow in the microfluidic structure with the fixed dimension of the gap in the trap and the different width of the main channel. With the increase of the channel width, the flow rate in the main channel increases while the flow rate decreases in the trap. If the channel width is extremely large with respect to the dimension of the gap, the flow directly passes through the main channel and no flow enters the trap. Therefore, the width of the main channel should be comparable to that of the gap to ensure that the hydraulic resistance in the channel is similar to that in the trap so as to realize a balance in the flow division.

In the design of the microfluidic structure, the dimension of the gap in the trap should be slightly smaller than the size of a single cell. There is a compromise in the determination of the width of the main channel. A wide main channel is preferred for a high throughput and also to prevent itself from being blocked by large cells or cell clusters. Nevertheless, the main channel cannot be so wide as to cause imbalance in the flow division. Figure S4 demonstrates the flow in the microfluidic structure with an extremely wide main channel (140 μm) and a *w/s* of 14. The flow is only in the main channel and does not enter the trap because the hydraulic resistance in the main channel is much lower than that in the trap. The air cannot be extruded out of the trap and micro-bubbles are stranded in the traps. The height of the microstructure is suggested to be 2–3 times the average size of the cells to allow cells to easily pass through and efficiently block the gap once the trap is occupied.

### 3.3. Demonstration of Cell Trapping

**Figure 7:**
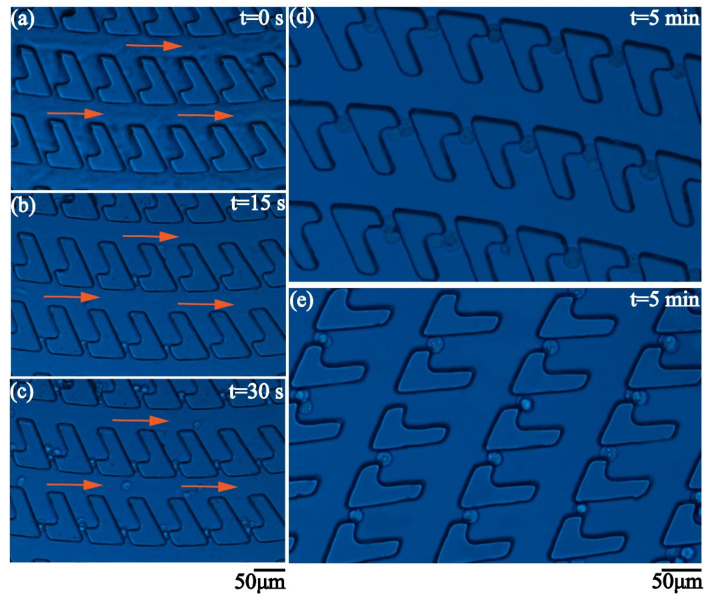
(**a**–**c**) Time-lapse montage of the procedure of cell trapping. (**d**,**e**) Microscopic images demonstrating that the traps in the different parts of the dentate spiral channel capture the 4T1 cells after 5 min.

In the demonstration of cell trapping, 2 × 10^5^ 4T1 cells are diluted in 10 mL phosphate buffer saline (PBS). Then, 200 μL of sample is injected into the microfluidic chip at the flow rate of 40 μL/min. The size of the 4T1 cells has the range of 8 μm to 23 μm. Microscopic images of the cells are illustrated in Figure S5. The gap is 10 μm and the width of the main channel is 30 μm. The density of the traps is 120 traps/mm^2^. Figure 7a–c show the procedure of the cell trapping. The flow is unidirectional from the inlet to the outlet. When coming across the trap, the cells flowing in the spiral channel suddenly make a turn and enter the empty trap. If the size of the cell is much less than the height of the channel, the cell cannot completely block the gap and multiple trapping happens. The following cells would enter the trap until the gap is blocked. Once the gap is almost completely blocked, the trap becomes a dead end. The following cells would pass by the trap and keep flowing in the main channel due to the extremely high hydraulic resistance in the trap. The concavity of the trap is large enough to store several single cells or a large cell cluster. The flow in the main channel keeps a high flow rate and has no influence over the cells captured in the traps. The cell trapping is very efficient. Figure 7d,e show the different parts of the dentate spiral channel when the injection lasts for five minutes. Every trap captures at least one cell. The captured cells stably stay in the traps.

**Figure 8:**
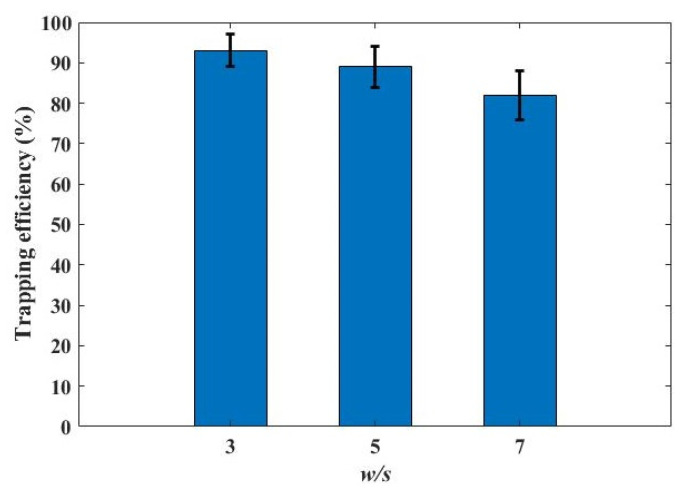
The influence of the ratio between the width of the main channel and the dimension of the gap, i.e., *w/s*, over the trapping efficiency.

Cell trapping efficiency is investigated in the dentate spiral channels with the gap of 10 μm and the main channels of 30 μm, 50 μm and 70 μm, i.e., *w/s* = 3, 5 and 7. The total number of the cells injected into the microfluidic chip is *n_Total_* and the number of the cells flowing out of the chip is *n_Out_*. The trapping efficiency is calculated as (*n_Total_–n_Out_*)/*n_Total_*. In the experiment, 4000 cells are counted by using a cell sorter (BD FACSMelody™ Cell Sorter) and injected into the chips. The cells flowing out of the chip are counted on a hemocytometer. Figure 8 shows the measured trapping efficiency. The error bars represent the deviation in the measurements for three times. In the dentate spiral channel with *w/s* = 3, a high trapping efficiency (>90%) is achieved. With the increase of *w/s*, the flow division to the main channel becomes dominant and the chance for the flow carrying the cells to enter the traps is reduced. As a result, the trapping efficiency drops. Thus, the dentate spiral channel with a very wide channel is not suggested for cell trapping.

## 4. Conclusions

In this study, we elaborate the operation principle, the equivalent circuit model and optimization of a cell-trapping microfluidic structure, which consists of a spiral main channel and an array of traps on the side of the channel. Since the traps are off the way of the main stream, cells move unidirectionally in the main channel at a high speed without any obstacle. When a cell or cell cluster comes across an empty trap, the cell would make a turn and enter the trap due to the flow division. If the gap in the trap is blocked by the cells, the mode of the trap turns into “closed” and it becomes hard for the following cells to enter the occupied trap due to the high hydraulic resistance in the trap. The structural design affects the flow. If the main channel is much wider in contrast to the gap in the trap, the flow keeps moving only in the main channel and does not go into the trap. To achieve high-throughput, high-efficiency cell trapping, a proper structural design is required to balance the hydraulic resistance in the main channel and the trap. Finally, the trapping of 4T1 mouse breast cancer cells, whose average diameter is about 15 μm, is demonstrated. The cells can be efficiently captured and isolated in the different traps. In the proposed microfluidic structure, the number of the traps is large and the density of the traps is high. It is feasible to capture a large number of cells in a single chip. The proposed cell-trapping structure has the potential to be applied in the various biomedical microfluidic applications, including cell-based assays, extraction of rare circulating tumor cells and drug screening.

## Data Availability

The data presented in this study are available on request from the corresponding author.

## Supplementary Materials

The following are available online at https://www.mdpi.com/2072-666X/12/3/288/s1, Figure S1: Model of the hydraulic resistance in the main channel and the trap, Figure S2: Model of the dentate spiral microfluidic channel used in the simulation, Figure S3: (a) Simulation of the flow in the structure with *w*/*s* = 3, when all the traps are empty. (b) Simulation of the flow in the structure with *w*/*s* = 3, when a part of traps are occupied. (c) The normalized pressure measured at different positions as marked in (a) and (b)., Figure S4: The flow in the dentate spiral channel with the 140 μm main channel and the 10 μm gap, Figure S5: The microscopic images of the 4T1 cells used in the cell-trapping experiment.
