# Supplementary material for: High-Throughput Cell Trapping in the Dentate Spiral Microfluidic Channel
Source: Micromachines (Basel). 2021 Mar 9;12(3):288. doi: 10.3390/mi12030288 (PMC8000121; doi:10.3390/mi12030288)
Supplement: Supplementary file 1 [file micromachines-12-00288-s001.pdf]

micromachines

MDPI

Supplementary Information: High-throughput cell trapping in the dentate spiral microfluidic channel

Jiawei Lu, Bo Dai, Kan Wang, Yan Long, Zhuoqing Yang, Junyi Chen, Shaoqi Huang, Lulu Zheng, Yongfeng Fu, Wenbin Wan, Songlin Zhuang, Yangtai Guan * and Dawei Zhang *

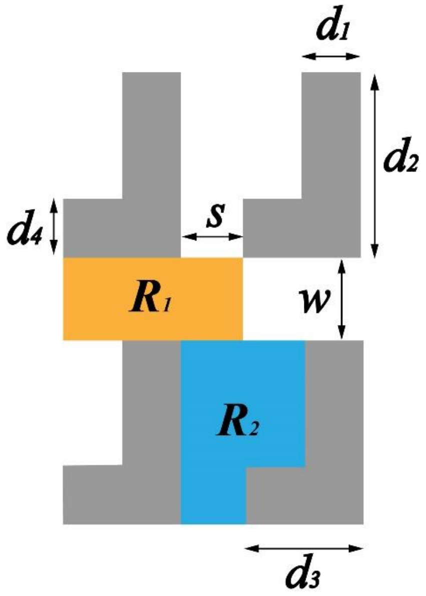

Figure S1. Model of the hydraulic resistance in the main channel and the trap.

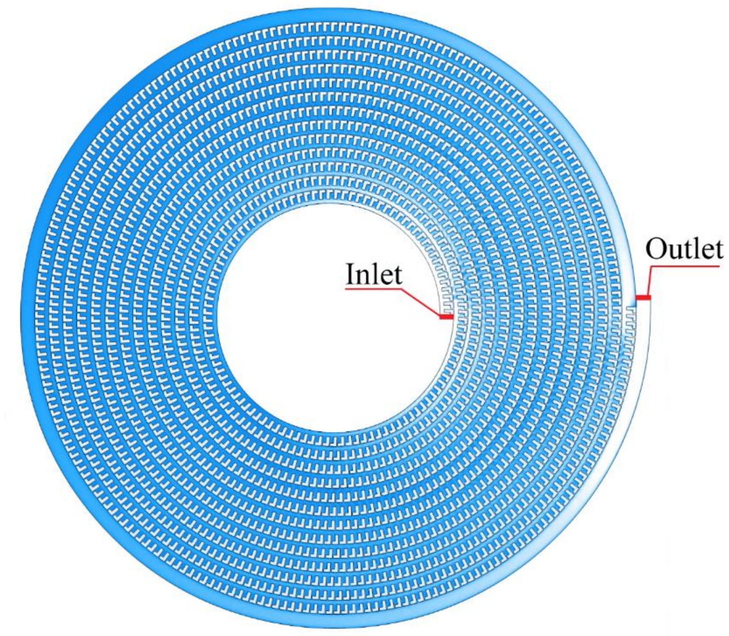

Figure S2. Model of the dentate spiral microfluidic channel used in the simulation.

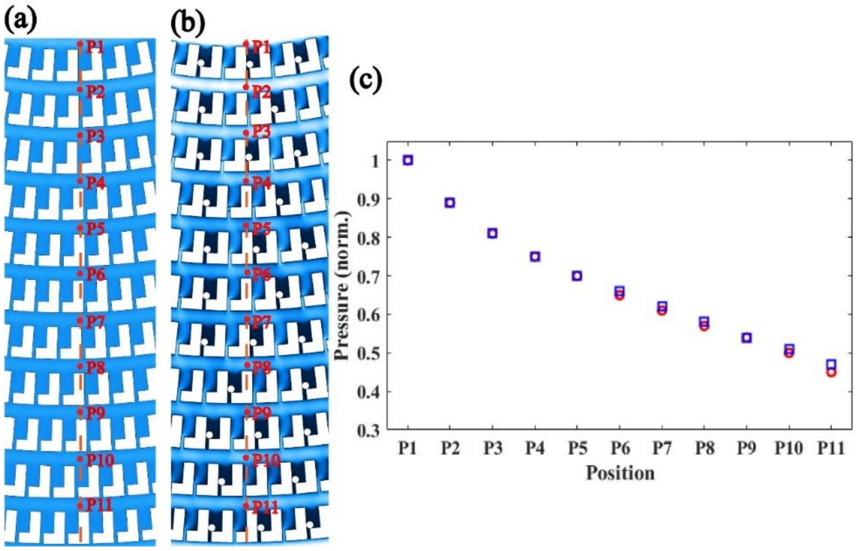

Figure S3. (**a**) Simulation of the flow in the structure with $w/s=3$, when all the traps are empty. (**b**) Simulation of the flow in the structure with $w/s=3$, when a part of traps are occupied. (**c**) The normalized pressure measured at different positions as marked in (**a**) and (**b**). Red circle: the normalized pressure when the traps are empty. Blue square: the normalized pressure when a part of traps are occupied.

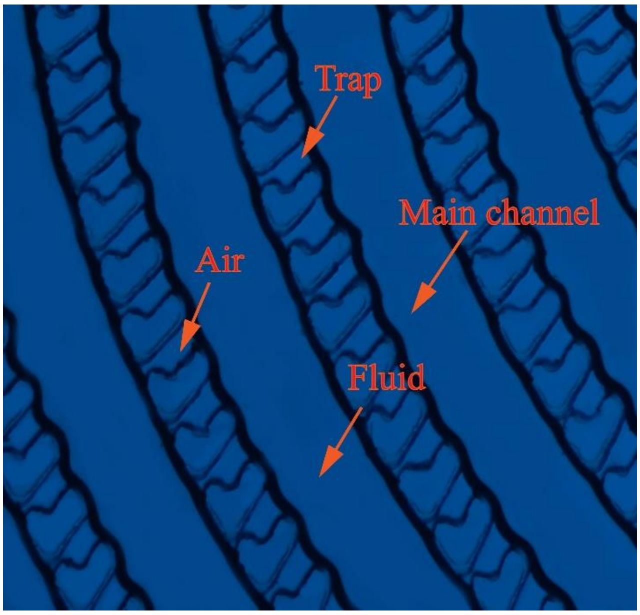

Figure S4. The flow in the dentate spiral channel with the 140 μm main channel and the 10 μm gap, *i.e.*, $w/s=14$. There is no flow division. The flow passes by the traps and does not enter the trap. The air is stranded in the traps.

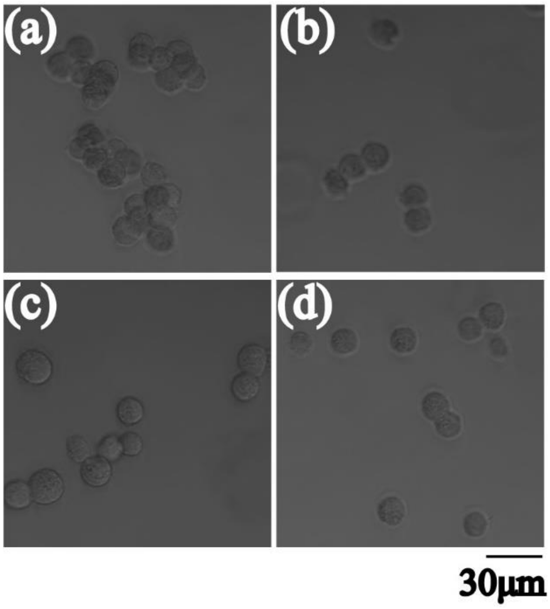

Figure S5. The microscopic images of the 4T1 cells used in the cell-trapping experiment.
